# Preventing children from drowning with AI algorithm in enclosed spaces: a review article

**Published:** 2023-12

**Authors:** Seif Askary Samira, Sima Seif Askary

**Affiliations:** ^*a* Department of Information Technology, Shahid Beheshti School of Nursing and Midwifery, Guilan University of Medical Sciences, Rasht, Iran. ^; ^*b* MA in Economics, University of Isfahan, Isfahan, Iran. ^

## Abstract

**Background:**

Drowning is one of the leading causes of death from unwanted injuries in the world. The highest death rate from drowning is among children. Pools are one of these places that have significant problems in current systems due to technical and methodological aspects. Using an automated visual monitoring system can help reduce drowning. The main objective of the project is to minimize the death of children from drowning.

**Methods:**

This research was performed using applied research and content analysis methods and through up-to-date international studies. In this study, cameras were installed around swimming pools. A threshold surface is defined for the depth of water. Images of children are taken every time they enter the pool and stored in databases. If a child reaches below the depth of the threshold, the child is identified and a warning message is sent to the rescuer. The name of the drowned child will be announced and action can be taken to save the child.

**Results:**

The presented algorithm can detect drowning conditions along with the exact position of the person drowning in the pool and provide the result in the best possible time, which is relatively low compared to the rescue time required for the drowning rescue operation.

**Conclusions:**

In order to solve the problem of insufficient monitoring, the child drowning prevention system was examined. Implementing the algorithm under study makes rapid decision-making possible. The proposed system has shown that it can be implemented in domestic swimming pools and public pools to prevent unintentional drowning deaths. The survey is a method of tracking humans within the framework of a video surveillance system capable of automatically detecting drowning in a pool.

**Keywords:**

Artificial intelligence, Drowning, Image processing

